# Current Perspectives on Viable but Non-Culturable Foodborne Pathogenic Bacteria: A Review

**DOI:** 10.3390/foods12061179

**Published:** 2023-03-10

**Authors:** Jiawen Zhang, Haoqing Yang, Jing Li, Jiamiao Hu, Guanyuan Lin, Bee K. Tan, Shaoling Lin

**Affiliations:** 1 College of Food Science, Fujian Agriculture and Forestry University, Fuzhou 350002, China; 2 Department of Cardiovascular Sciences and Diabetes Research Centre, University of Leicester, Leicester LE1 7RH, UK

**Keywords:** foodborne pathogens, VBNC, induction, detection, formation mechanism

## Abstract

Foodborne diseases caused by foodborne pathogens pose risks to food safety. Effective detection and efficient inactivation of pathogenic bacteria has always been a research hotspot in the field of food safety. Complicating these goals, bacteria can be induced to adopt a viable but non-culturable (VBNC) state under adverse external environmental stresses. When in the VBNC state, pathogens cannot form visible colonies during traditional culture but remain metabolically active and toxic. The resulting false negative results in growth-related assays can jeopardize food safety. This review summarizes the latest research on VBNC foodborne pathogens, including induction conditions, detection methods, mechanism of VBNC formation, and possible control strategies. It is hoped that this review can provide ideas and methods for future research on VBNC foodborne pathogenic bacteria.

## 1. Introduction

Foodborne diseases caused by the ingestion of various pathogenic bacteria are a major global public health and food safety issue. Particularly, the disease burden (both epidemiological and economic) associated with the occurrence of foodborne diseases requires significant national resources to address [1,2]. The World Health Organization reported on 7 June 2021 that approximately 600 million cases of foodborne illness occur worldwide annually [3]. Bacterial foodborne illnesses cause the majority of foodborne illnesses in both developed and developing countries [4]. For example, 20,017 cases of foodborne illness occurred in 27 European Union countries in 2020, with *Campylobacter* being the cause of more than 60% of confirmed cases. Other common foodborne pathogens include *Salmonella* and *Yersinia* [5]. In Africa, nearly 70% of foodborne illnesses are caused by bacteria (mainly *Salmonella* and enteropathogenic *Escherichia coli*) each year [1]. In addition, other pathogenic bacteria commonly found in various regions include *Vibrio parahaemolyticus* and *Shigella* [1]. Generally, these pathogenic bacteria cause typical intestinal symptoms, such as vomiting, diarrhea, and abdominal pain; in more severe cases, the symptoms can be life-threatening [6]. Therefore, inactivation and detection of viable pathogenic bacteria in food are important to ensuring food safety and human health.

Plate culture is one of the most common conventional bacterial detection methods [7]. The method relies on the growth of viable bacteria on a solid medium. The number of generated colonies is counted to obtain the number of viable bacteria in the sample. However, when faced with adverse environmental stress, bacteria can respond by entering a special dormant state, in which the bacteria are viable but non-culturable (VBNC) [8].

**Figure 1:**
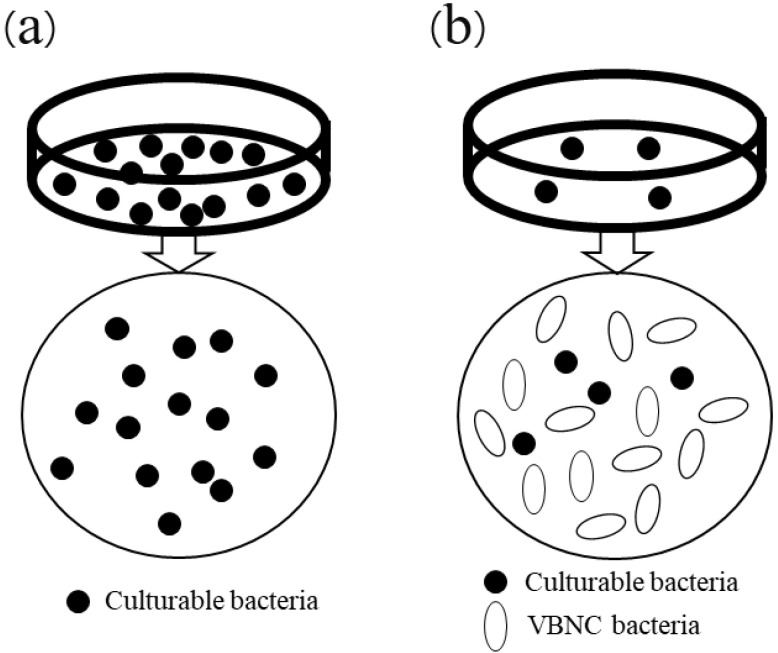
Schematic diagram of culturable bacteria (**a**) and VBNC bacteria (**b**).

The phenomenon of VBNC bacteria was first proposed and supporting evidence was presented in 1982 by a team of researchers headed by Professor Xu and Rita Colwell. Bacteria in the VBNC state retain intact membranes, undamaged genetic material, and are still metabolically active, and, if pathogenic, remain virulent. It has been shown that in the presence of an inhibitor of DNA synthesis and defined quantities of certain nutrients, bacteria elongate and grow, but not to the extent necessary to form visible colonies. Even though viable, these bacteria are not detectable in traditional colony-related assays. Instead, as bacteria enter the VBNC state, the viable count decreases rapidly, artifactually indicating a loss of viability. Thus, the credibility of the plate detection assay is reduced (Figure 1) [8]. When conditions are more hospitable, the bacteria can recover from the VBNC state. If this occurs in food, bacterial foodborne diseases can result [9].

More than 100 kinds of bacteria can enter the VBNC state. These include many foodborne pathogenic bacteria, such as *Staphylococcus aureus*, *Escherichia coli O157:H7*, *Salmonella*, and others [9]. The bacteria pose a considerable (and hidden) danger in food safety assessment. This paper reviews the current understanding of the induction, detection, control, and mechanisms of the formation of the VBNC state. The information provides a theoretical basis and reference for future research on the VBNC status of various pathogenic bacteria in food.

## 2. Foodborne Pathogenic VBNC Bacteria and Induction Conditions

Initially, the VBNC state was observed in *Vibrio cholerae* and *E. coli* present in marine and estuarine environments [8]. Since then, a variety of bacteria capable of VBNC existence have been detected in different foods, including vegetable products, fruits, meat products, dairy products, rice and flour products, and tea and related products. The conditions that can induce foodborne pathogenic bacteria to adopt the VBNC state have been extensively studied, and a series of discoveries have provided important clues for an in-depth understanding of the mechanisms of action and metabolic characteristics of VBNC bacteria.

Table 1 summarizes the common foodborne pathogenic bacterial species that can become VBNC and their induction conditions. In general, adverse external conditions, including nutrient starvation, low temperature, acid treatment, high salt treatment, oxygen stress, and others, are important for the induction of VBNC. For example, nutrient starvation treatment of *S. aureus* revealed that colonies enter the VBNC state within a short period of time [10]. These bacteria become VBNC even faster with a hostile pH environment or salt concentration. Concerning food, certain physical treatments and chemical additives during food processing and storage might also be responsible for inducing the self-protection mechanism of bacteria in food. For instance, a range of non-thermal food sterilization treatments including high hydrostatic pressure, pulsed light, ultraviolet (UV) radiation, and cold plasma treatment [11] have been found to pose the risk of inducing the entry of bacteria into the VBNC state. Kramer B et al. reported that pulsed light with a broad spectrum between 200 and 1100 nm produced by the capacitor with 1–3 kV could effectively kill various microbial pathogens and denature foodborne species in food. However, this approach was also found to induce *E. coli* to enter the VBNC state [12].

## 3. VBNC Entry Mechanisms of Foodborne Pathogens

### 3.1. Stringent Response

**Figure 2:**
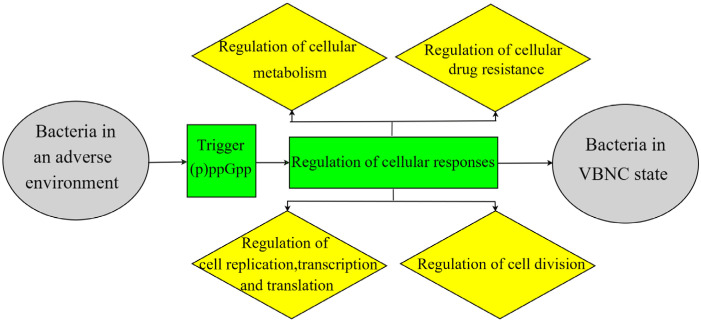
Schematic diagram of stringent response theory.

The stringent response is a key survival mechanism often used by bacteria in response to adverse environmental conditions [37], which is elicited through the synthesis of a small-molecule alarmone (guanosine pentaphosphate; (p)ppGpp). (p)ppGpp is a typical alarm factor responsible for sensing environmental stresses and triggering a stringent response. Accordingly, (p)ppGpp re-intervenes the cellular response machinery, inducing a downstream pathway to drive the bacteria into a dormant state. Bacterial (p)ppGpp regulates replication, transcription, and translation, and affects bacterial physiological states through binding to several cellular targets (e.g., RNA polymerase) and through regulatory pathways related to metabolism, cell division, and antibiotic resistance. (p)ppGpp-mediated stringent responses may play an important role in the entry of bacteria into the VBNC state (Figure 2) [38,39]. The synthesis and hydrolysis of (p)ppGpp depend on the activity of two proteins, RelA and SpoT. Elevated activity of the *relA* and *spoT* genes upon entry into the VBNC state leads to the accumulation of (p)ppGpp, resulting in the enhancement of stress resistance [40].

### 3.2. Toxin–Antitoxin (TA) System

**Figure 3:**
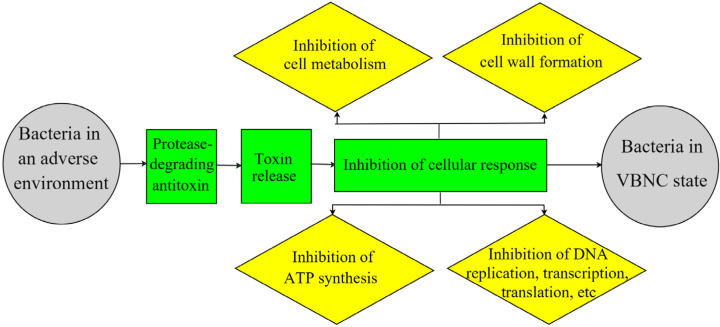
Schematic diagram of toxin–antitoxin (TA) system theory.

Bacteria are characterized by a complex of stable toxin proteins that slow bacterial growth or directly mediate bacterial death, and unstable antitoxin proteins that inhibit the expression of toxin proteins. The proteins are controlled by plasmid- or chromosome-encoded TA genes [41]. The TA system is activated when bacteria are exposed to adverse external environmental stresses. The system uses the differential expression of TA to regulate bacterial self-screening for survival adaptation. This is evidenced by the fact that unstable antitoxins are degraded by proteases, while the simultaneous expression of toxin proteins regulates the reduction of metabolic turnover by inhibiting essential cellular processes, such as DNA replication, translation, protein synthesis, ATP synthesis, and cell wall formation, causing the cell to enter a VBNC dormant state [41,42]. The expression levels of genes related to the TA trigger system (*rpoS*, *spoT*, *relA*, *ppx*, *ppk*, and *lon*) as well as the antitoxin proteases Lon and ClpP are upregulated in bacteria after induction of the VBNC state upon exposure to an adverse environment (Figure 3) [43,44].

### 3.3. Oxidative Stress

Increased oxidative damage and oxidative stress are observed upon entry of bacteria into the VBNC state, as evidenced by an increase in the production of reactive oxygen species (ROS), including superoxide anion, hydrogen peroxide, and hydroxyl radicals [45]. For instance, a previous study revealed that the production of the free radicals (carbon-centered (ethanol) radicals, hydroxyl radicals, and hydrogen protons) displays a significant non-linear sigmoidal curve relationship with the VBNC incidence index when VBNC is induced in *Salmonella* by thermo-sonication. This provided the first quantitative data supporting the involvement of ROS in VBNC induction [46]. In addition, pretreatment of *Salmonella* with sodium pyruvate radical scavenger before thermal sonication reportedly inhibited VBNC production, again verifying that free radicals were an important influencing factor in the formation of VBNC bacteria [46]. Bacterial oxidative stress defense systems, which usually include alkyl hydroperoxide reductase (AhpC), catalase (KatA, KatG, and KatE), and superoxide dismutase (SodA, SodB, and SodC), could balance internal oxidation [47,48]. Experiments using three *Campylobacter jejuni* mutants defective in key antioxidant genes (including *ahpC*, *katA*, and *sodB*) were performed to study the effect of oxidative stress resistance on bacterial survival under aerobic conditions. The three mutants displayed reduced viability and morphological changes exclusive to VBNC bacteria. When antioxidant treatment was restored, the number of colonies entering the VBNC was relatively reduced [47]. The findings clearly indicate that oxidative stress has an important role in the induction of VBNC bacteria. The exact molecular mechanism remains unclear and needs further investigation.

### 3.4. Gene Regulation

The mechanism of bacterial entry into VBNC involves complex processes which cannot be separated from the regulation of genes. RNA sequencing (RNA-Seq) transcriptomics and isobaric tag for relative and absolute quantitation (iTRAQ) proteomic methods have been used to compare transcriptional and protein expression differences in *E. coli O157:H7* in the normal and VBNC states. The authors identified 97 genes and 56 proteins that were significantly altered upon entry into the VBNC state, which mainly involved genes and proteins related to membrane transport, metabolism, DNA replication, and cell division, and which led the cells to the VBNC state of low metabolic activity [49]. Similarly, genomic characterization of VBNC *V. cholerae* revealed changes in the expression of 1420 genes, with upregulation of genes related to biofilm formation and stress response, and downregulation of genes related to cell division and ribosome activity [50]. A total of 16 genes were significantly upregulated when *E. coli* was in the VBNC state, including stress response-induced genes, three genes encoding toxic proteins (*ygeG*, *ibsD*, and *shoB*), and others [17]. Taken together, the gene expression analysis provided novel insight into the regulation mechanisms of bacteria entering into the VBNC state when exposed to a harsh environment.

### 3.5. Regulation of Protein Aggregation

**Figure 4:**
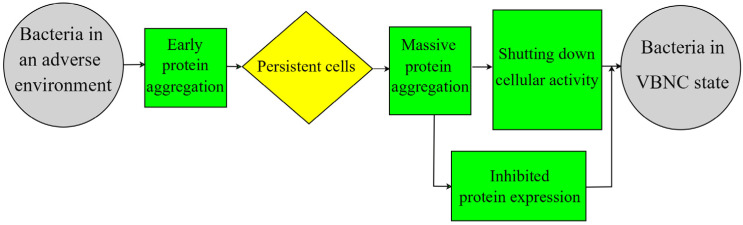
Schematic diagram of protein aggregation regulation theory.

Bacteria can become persistent cells, which is defined as a dormant, non-dividing, and metabolically inactive state. Indeed, bacteria in the VBNC could be considered as a deeper level of dormancy. Since persistent and VBNC cells are very similar in their dormancy and tolerance of high antibiotic concentrations, the increasingly accepted view is that persistent and VBNC cells might represent different stages of the same dormancy process [51]. During this process, the appearance and increase in protein aggregates correlate with bacterial persistence [52]. This has prompted investigations of the role of protein aggregation in the dynamic transition of bacteria from the persistent to the VBNC state. Induction of protein aggregation was observed as bacteria progressively entered dormancy, with persistent cells starting to produce protein aggregates at an early stage and VBNC cells harboring more mature protein aggregates (Figure 4) [53,54]. Since the aggregates contained many proteins required for translation, researchers have also theorized that the massive aggregation of these proteins shuts down cellular translational activity and thus induces dormancy. Furthermore, researchers have also found that the expression of Obge, a persistent protein that interacts with ribosomes, could exacerbate protein aggregation and facilitate the transition to the dormant state. These findings coincide with the results of Anusuya et al. [55] on the proteomic characterization of VBNC and recovered *V. cholerae*, where 19 proteins related to carbohydrate metabolism, phosphate utilization, and stress response appeared to be highly expressed when bacteria recovered from the dormant state. 

### 3.6. ATP Regulation

**Figure 5:**
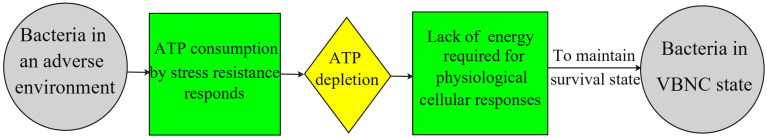
Schematic diagram of ATP regulation theory.

As mentioned above, the depth of dormancy of bacterial cells has been closely related to the appearance of a series of cellular features caused by protein aggregation, with the formation of cellular protein aggregates facilitating cellular dormancy. Notably, the dynamic protein mass control system in bacterial cells was shown to be highly dependent on ATP, such that ATP can be used as a biological hydrotrope to maintain proteolysis and prevent macromolecular aggregation [56]. Protein aggregation was observed when ATP was depleted [54]. Liao et al. [16] found that non-thermal plasma (NTP) induced *S. aureus* to enter the VBNC state, where the oxidative stress generated by NTP triggered an oxidative stress response in the bacterium, which consumed part of the cellular ATP and made other energy-dependent physiological activities (e.g., cell growth and division) less energetic (Figure 5). These events forced bacteria into the VBNC state, contributing to the long-term maintenance of cell viability. Notably, the variation in ATP content is dependent on various stress factors and bacterial species.

## 4. Detection of VBNC Foodborne Pathogenic Bacteria

The traditional plate counting method only enumerates culturable bacteria. VBNC microorganisms cannot grow and reproduce on a culture medium to form visible colonies, and so cannot be enumerated by plate counting. This is also one underlying reason why foods with colony-forming units in the acceptable range still cause foodborne bacterial illnesses. Currently, the enumeration of viable bacterial counts relies mainly on a variety of physiological characteristics of VBNC bacteria. These characteristics include oxidative respiratory activity determined by 5-cyano-2,3-ditolyl tetrazolium chloride (CTC) or iodonitrotetrazolium chloride (INT) staining, substrate uptake determined by direct viable counting (DVC), membrane permeability (SYTO 9/propidium iodide (PI) staining), nucleic acid detection (propidium monoazide–quantitative polymerase chain reaction, PMA-qPCR), and RNA expression (RT-qPCR) [23]. The following paragraphs summarize commonly used methods for the detection of VBNC microorganisms.

### 4.1. Staining or Fluorescent Labeling

#### 4.1.1. Redox Test Method

The redox test method is based on the fact that VBNC bacteria retain low respiratory activity and can reduce a specific dye to a fluorescent product. When the dye enters the cell, it can be reduced by respiratory chain enzymes of VBNC bacteria to produce a red fluorescent precipitant. The fluorescence can be observed and quantified by fluorescence microscopy or flow cytometry. The typically used dyes include CTC or INT [57,58,59]. For instance, Zhu et al. [60] successfully quantified the number of *E. coli* in the VBNC state after UV treatment using CTC flow cytometry. The number of *E. coli* entering the VBNC state was proportional to the germicidal power in the UV range of 0–30 mJ/cm^2^. This is a relatively simple detection method. However, the toxicity of CTC can lead to an underestimation of the level of active cells, and stained bacteria grow at the same rate as untreated bacteria only at low concentrations of the dye [58].

#### 4.1.2. DVC

The DVC method was proposed in 1979. Nalidixic acid is a DNA helicase inhibitor. When nalidixic acid was added to Gram-negative (G−) bacteria samples, viable bacteria were inhibited from dividing with the morphology changed to become significantly longer and thicker. When the samples were further stained with acridine orange, live bacteria could be distinguished by fluorescence microscopy based on the altered morphology of the viable bacteria. Nalidixic acid is less effective in inhibiting Gram-positive (G+) bacteria, so the division inhibitor used for the DVC test for G+ is often ciprofloxacin [57,61]. This method has been applied to many viable bacteria, such as *Listeria monocytogenes* [57], *V. cholerae* [62], and *S. typhi* [13]. Besnard et al. used ciprofloxacin to stain and incubate starved *L. monocytogenes* and observed the normal morphology of dead cells versus the elongated morphology of live cells by fluorescence microscopy. The proportion of live cells was determined by random counting of multiple microscopic fields [57]. Similarly, different samples containing *V. cholerae* were measured by the conventional culture method versus ciprofloxacin by fluorescence microscopy. Only 15.11% of the samples were positive for *V. cholerae* in the conventional culture method, whereas 40.70% of the samples were positive for *V. cholerae* using the DVC live microscopy assay, showing the accuracy of the measurement by DVC microscopy [62]. This is an easy-to-operate assay. The influential factor is the difference in the inhibitory effect of different DNA division inhibitors on bacteria.

#### 4.1.3. Cell Membrane Permeability Test Based on Fluorescent Dyes

The use of fluorescent dyes to examine cell membrane permeability can also identify and quantify bacteria in the VBNC state. The most commonly used fluorescent dyes are SYTO 9 and PI. SYTO 9 is able to penetrate the cell membranes of both living and dead cells to bind DNA and RNA, resulting in green fluorescence. PI is only able to penetrate dead or damaged cells, resulting in red fluorescence [63]. Differences in fluorescence colors enable the differentiation of living and dead cells. For example, Deng et al. evaluated the optimal dye concentration for a SYTO 9/PI kit by flow cytometry and proposed the optimal staining parameters for *Bifidobacterium* [63]. Zhu et al. used SYTO 9/PI staining in combination with flow cytometry to analyze the effect of different concentrations of NaClO on *E. coli* entry into the VBNC state. The authors measured the percentage of live cells and demonstrated that elevated NaClO concentrations accelerated entry into the VBNC state [60]. The SYTO 9/PI assay is simple to perform and rapid. However, since SYOT 9 and PI fluorescence signals are affected by fluorescence resonance energy transfer, background signals, and binding affinity, high amounts of dye can alter membrane properties and promote membrane transport, which may lead to greater errors [63,64]. In addition to SYTO 9/PI staining, other fluorescent dyes have been successfully applied to quantify VBNC bacteria. For example, carboxyfluorescein diacetate (cFDA) is a non-fluorescent precursor that readily diffuses across the cell membrane. Upon internalization, it hydrolyzes the diacetate groups to the membrane-impermeant fluorescent compound cF as catalyzed by a non-specific esterase. cFDA can be used as a substitute for SYTO 9 [65].

### 4.2. Molecular Biological Test Methods

#### 4.2.1. PMA-qPCR

The nucleic acid binding dyes ethidium monoazide (EMA) and PMA can enter dead cells with damaged cell membranes. Both dyes covalently cross-link to the dead cell DNA, thereby reducing or eliminating the amplification of DNA fragments in the subsequent PCR in dead cells, leaving live cells unaffected. The distinction permits a reasonably accurate estimation of the number of live cells. EMA is sometimes able to penetrate through live or intact bacteria, leading to false negative results, so the most commonly used nucleic acid amplification inhibitor is PMA [66]. Liang et al. established a PMA-qPCR system for the detection of *S. typhimurium* in lettuce. After incubating the samples with PMA dye, the *invA* gene was selected as the target gene for amplification. Typically, the final minimum detection limit for *Salmonella* in lettuce was 10^3^ CFU/g. The detection limit could be reduced to 10^1^ CFU/g when a 12 h bacterial enrichment was performed [67]. This technique greatly reduces the detection time and is very accurate. However, expensive analytical equipment is required.

#### 4.2.2. PMA-LAMP

LAMP is an automated cyclic DNA synthesis method that does not require complex thermal cycling apparatus and can amplify target DNA within 1 h at constant temperature (60–65 °C) using four to six specially designed primers and a strand-displacing DNA polymerase [68,69]. Liu et al. selected the *wzy* gene of *E. coli O157:H7* and *agfA* of *S. enterica* for LAMP and quantitatively determined VBNC bacteria in different agricultural products that included tomato and spinach. The detection limits for *E. coli* and *S. enterica* were 5.13 × 10^3^ and 1.05 × 10^4^ CFU/g, respectively. Peterse et al. developed a PMA-LAMP assay targeting the *hipO* gene of *C. jejuni*. The assay had a 100% targeting specificity for this bacterium and was successfully used for the quantification of VBNC *C. jejuni* in agricultural products that included chicken breast meat and romaine lettuce. The detection limits were as low as 10^2^ CFU/g [70]. Compared with PCR, the rapid LAMP detection technique permits simpler and faster nucleic acid amplification. Furthermore, the specific target DNA amplification can be performed in one step, without the need for advanced instruments.

#### 4.2.3. RT-qPCR

The large double helix structure can reduce the measurement sensitivity. Single-stranded mRNA is a more suitable target than DNA to assess cell viability by virtue of its small molecular weight, half-life of only a few minutes, and presence only in living cells. In the RT-qPCR measurement of cellular mRNA, the mRNA is reverse transcribed to cDNA, which is used as a template for PCR amplification. In one study, *Salmonella* in milk samples was quantified based on RT-qPCR *Salmonella invA* mRNA. The method had a minimum measurement limit of 10^4^ CFU/mL for *Salmonella* and sensitivity up to 10^1^ CFU/mL after 12 h of bacterial enrichment [71]. The RNA-based method for the detection of live bacteria has more potential than the DNA-based approach, although mRNA extraction is challenging [71].

### 4.3. Novel Test Methods

#### 4.3.1. Raman Spectroscopy

**Figure 6:**
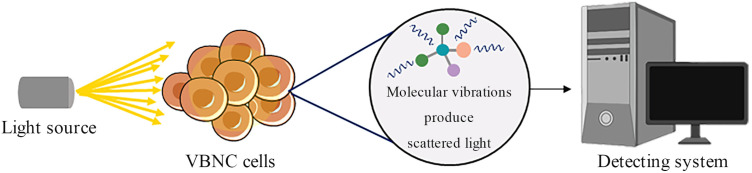
Schematic diagram of Raman spectroscopy.

The beam generated by Raman spectroscopy produces scattered light when it irradiates the detectors. The system receiving the scattered signal provides a comprehensive intracellular molecular spectrum based on the vibrational frequencies of the characteristic chemical bonds. The spectral patterns produced by the detectors are analyzed and identification is based on spectral characteristics that include the number and length of the spectral lines [72,73]. The detection of bacteria by Raman spectroscopy is mainly based on the quantification of cellular metabolic activity, where water molecules (H_2_O) actively participate in cellular metabolic activities, including the synthesis of fatty acids, proteins, and nucleic acids. The heavy hydrogen isotope deuterium (D) can replace H) in water molecules during fatty acid and protein synthesis to form carbon–deuterium (C–D) bonds. The integrated spectral intensity of C–D and C–H segments is detected using spectroscopy analysis to quantify the degree of substitution of H by D, and thus evaluate the metabolic activity of bacteria (Figure 6) [74,75]. Guo et al. [74] used Raman spectroscopy to measure the metabolic activity of four strains of *E. coli* and *S. aureus* upon entry into the VBNC state under UV irradiation. Decreased metabolic activity was evident. However, even with extremely high UV intensity, some metabolic activity was retained, which included transcriptional, translational, and virulence factor expression pathways [74]. Other assays are more concerned with the average metabolic activity of the cell population. By contrast, Raman spectroscopy can provide a comprehensive description of the metabolic activity of a single cell. However, Raman spectroscopy is more expensive and requires operator expertise, which limits its large-scale application.

#### 4.3.2. Biomarker Product Testing

**Figure 7:**
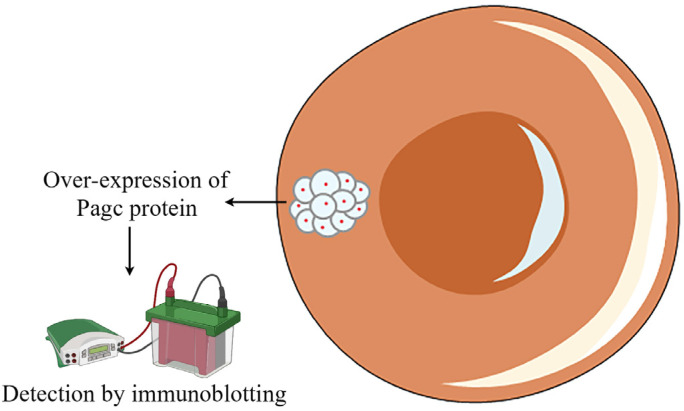
Schematic diagram of the biomarker testing method (protein).

The physiological response of the bacteria when they enter the VBNC state is altered. This is reflected in the different products produced by VBNC bacteria compared to their culturable counterparts. This difference can be exploited to detect VBNC bacteria. Jun et al. [76] chemically induced the VBNC state in *Salmonella*. The bacteria retained their shape but released many membrane vesicles accompanied by transient overexpression of membrane vesicle protein PagC. This protein could be used as a biomarker to detect VBNC *Salmonella* cells by immunoblotting (Figure 7). The assessment of VBNC *V. cholerae* revealed different trends of the up- and downregulation of different proteins, which might permit the detection of VBNC *V. cholerae* [55]. The many biomarkers that have been identified include proteins, nucleic acids, polysaccharides, phospholipids, lipids, and others. In addition, some specific chemicals are present only in particular microorganisms. However, the use of biomarkers in the detection of VBNC bacteria is presently hampered by the physiology of different colonies.

## 5. Control of Foodborne Pathogenic Bacteria in the VBNC State

VBNC bacteria can recover when cultured under certain conditions. Particularly, the recovery of toxin-producing bacteria in foods can result in damage to the human body, necessitating the control of these bacteria [77]. The comprehensive literature that has been amassed has identified two main strategies for achieving control of VBNC foodborne pathogenic bacteria. One strategy is to induce the direct death of microorganisms to avoid their entry into the VBNC state. The other is to control the recovery of microorganisms in the VBNC state and avoid new infections. Environmental factors are influential in the induction of VBNC. Thus, control of bacteria entering the VBNC state, and their subsequent recovery, can involve adjusting the physical and chemical conditions related to the microbial growth environment.

Several factors affect the resuscitation of bacteria from the VBNC state. These include temperature, humidity, oxygen concentration, acidity, and others. Wong et al. reported that a temperature upshift from 4 °C to 25 °C successfully recovered the cultivability of VBNC *V. parahaemolyticus* in a minimum salt medium. Interestingly, the upshift of the temperature to 37 °C instead of 25 °C significantly impaired the resuscitation [78]. The authors speculated that the increase in temperature during resuscitation may induce greater production of free radicals by the bacteria, resulting in lethal or sublethal damage to the bacteria. Optimization of temperature increase during the thawing of foods might be a promising strategy for minimizing the resuscitation of VBNC bacteria.

Besides physical conditions, bactericides that include antibiotics and chlorine can block entry into the VBNC state and can effectively decrease the viability of the VBNC bacteria. Truchado et al. evaluated the effect of chlorine on *L. monocytogenes* and *E. coli O157:H7* in process wash water. The authors reported that both bacteria were induced to enter the VBNC state after treatment with 2.5 mg/L chlorine. All *L. monocytogenes* cells were inactivated after treatment with 5 mg/L chlorine, but *E. coli O157:H7* entered the VBNC state [79]. The authors further increased the free chlorine concentration to 20–25 mg/L; this effectively inhibited the growth of both bacteria in process wash water, with no VBNC detected [80]. The findings indicate that chlorine treatment can achieve the effective inactivation of bacteria. However, the optimal treatment concentration must be optimized based on factors such as contact time and scale of operation. The addition of antibiotics can also inhibit the recovery of VBNC bacteria. As an example, *S. aureus* was induced to the VBNC state due to oxidative stress at a low temperature of 4 °C, followed by the addition of chloramphenicol. The recovery of the bacteria was completely lost because the antibiotic inhibited the synthesis of VBNC bacterial proteins [81]. The same experimental results were obtained with the addition of chloramphenicol in a *V. parahaemolyticus* recovery experiment [78]. Similarly, 100% inhibition of bacterial recovery was achieved after treatment with high doses of benzylpenicillin, piperacillin, and gentamicin for the VBNC state of *Enterococcus faecalis*. Benzathine and piperacillin can bind to penicillin-binding proteins that are required for the final stage of peptidoglycan synthesis in bacteria, resulting in the loss of enzymatic activity. Gentamicin inhibits protein synthesis [82].

## 6. Conclusions and Outlook

The diversity of bacterial species, their different environments, and the development and refinements of genomics and proteomics analyses have provided a wealth of data on VBNC bacteria. Recent research has increasingly addressed VBNC foodborne bacteria, with the emergence of new sterilization technologies that include pulsed light and neutral electrolyzed water sterilization technology, the development of more accurate and diverse detection technologies/instruments for VBNC bacteria, and the deeper exploration of the mechanisms related to the induction and control of VBNC bacteria.

Since the proposal of the VBNC bacteria state 40 years ago, research in a variety of scientific disciplines has enriched the understanding of VBNC bacteria. Future explorations and refinements will involve five aspects. First, new sterilization methods will mainly focus on the creation of environmental stresses that will act to sterilize samples. Since this may induce VBNC bacteria, conditions that induce the VBNC state and their mechanisms of action will be explored in-depth. Second, the effects of different food systems and different food processing conditions on the production of VBNC bacteria will be systematically explored to determine if there is a universal mechanism of food processing conditions that induce VBNC bacteria. Third, food safety researchers will continue to develop and refine accurate quantitative detection technologies for novel VBNC bacteria. Indeed, the detection methods discussed above (Table 2) have their own advantages and disadvantages, which should be selected according to different detection settings. Fourth, genes, proteins, and treatment conditions that differ between VBNC and normally growing bacteria will be increasingly characterized. Elucidation of the roles of these factors will be beneficial for the control and detection of VBNC bacteria. Finally, novel culture methods will continue to be developed to replace traditional culture methods that are not able to detect all foodborne pathogens in their entirety. Although nucleic acid and antigen-based detection of pathogens are currently available and are undeniably valuable, these techniques are not universally applicable. The present review provides a foundation for these research goals.

## Figures and Tables

**Table 1:** Common foodborne pathogenic bacteria that can enter VBNC and their induction conditions.

Bacterial Genus	Bacterial Strain	Bacterial Culture Medium	Induction Conditions	Common Food Species	Reference
*Salmonella*	*Salmonella* *typhimurium*	Sterilized water, beef peptone yeast broth/apple/carrot juice/physiological saline/phosphate buffer solution	−20 °C/CuSO_4_, thermo-sonication of 380 W at 53 °C for 30 min	Animal foodstuff	[13,14]
	*Salmonella* *enterica*	Culture medium	NaCl/peracetic acid/hydrogen peroxide at 4 °C		[15]
*Staphylococcus*	*Staphylococcus aureus*	Tryptic soy broth	Low-temperature treatment/nutrient starvation treatment/acid treatment, nonthermal-plasma treatment	Dairy, meat, starchy food (rice and flour products, leftovers), etc.	[10,16]
*Escherichia*	*E. coli (ATCC 25922)*, *E. coli DSM 498*	Culture solution	Pulsed light, low level chlorination, and atmospheric pressure plasma jet	Meat, dairy products, vegetables, marine products, etc.	[12,17,18]
	*E. coli O157:H7*	Tap water, artificial soil; 0.85% NaCl with pH 3.0	Boiling and microwave treatment, low soil moisture, and high-pressure carbon dioxide		[19,20,21]
*Vibrio*	*Vibrio* *parahaemolyticus*	Sterile 3% NaCl	4 °C	Marine products	[22]
	*Vibrio cholerae*	Artificial seawater/c-di-GMP VacciGrade	4 °C		[23]
	*Vibrio vulnificus*	Artificial seawater (pH 4–7)	NaCl treatment at 4 °C		[24]
*Listeria*	*Listeria* *monocytogenes*	Brain heart infusion broth	Benzalkonium chloride	Meat, eggs, marine products, vegetables, etc.	[25]
*Campylobacte*	*Campylobater* *jejuni*	Mueller–Hinton Broth	4 °C treatment under aerobic conditions	Animal foodstuff	[26]
*Shigella*	*Shigella dysenteriae*	Deionized water	-	Cold dishes	[27]
*Proteus*	*Proteus mirabilis*	Deionized water	High and low osmotic pressure/acidic conditions	Animal foodstuff (cooked meat and visceral products)	[28]
*Clostridium*	*Clostridium perfringens*	Meat products	-	Animal foodstuff	[29]
*Bacillus*	*Bacillus cereus*	Meat products, milk	-	Meat products, dairy products, vegetables, rice noodles, rice, etc.	[29,30]
*Helicobacter*	*Helicobacter pylori*	Sterile lake water, drinking water, and natural fresh water	4 °C treatment in the dark, chlorine treatment	Contaminated water, milk, instant food, leftovers, etc.	[31,32,33]
*Yersinia*	*Yersinia pestis*	Artificial sea water	4 °C	Meat products, dairy products, drinking water, vegetables, etc.	[34]
	*Yersinia pseudotubercnlosis*	Liquid nutrient broth	-		[35]
	*Yersinia* *enterocolitica*	Trypticase soy broth	Neutral electrolyzed water		[36]

**Table 2:** Advantages and disadvantages of VBNC detection methods.

Detection Principal	Detection Methods	Advantages	Disadvantages
Staining or fluorescent labeling	Redox test method	Easy to operate	Toxic to active bacteria; flow cytometer or fluorescence microscope required
	DVC	Easy to operate	Fluorescence microscope required
	Cell membrane permeability test based on fluorescent dyes	Easy to operate	Flow cytometer or fluorescence microscope required
Nucleic acid amplification assay	PMA-qPCR	Easy to operate, fast detection, and high accuracy	qPCR thermocycler required
	PMA-LAMP	Easy to operate, fast detection, and simple equipment requirements	Difficulty in amplification primer design
	RT-qPCR	Easy to operate and fast detection	qPCR thermocycler required
Novel test methods	Raman spectroscopy	High accuracy; analysis possible at the single-cell level	Raman spectrometer required
	Biomarker product testing	High sensitivity	Limited to available biomarkers

## Data Availability

Not applicable.
